# Identifying Transmission Cycles at the Human-Animal Interface: The Role of Animal Reservoirs in Maintaining Gambiense Human African Trypanosomiasis

**DOI:** 10.1371/journal.pcbi.1002855

**Published:** 2013-01-17

**Authors:** Sebastian Funk, Hiroshi Nishiura, Hans Heesterbeek, W. John Edmunds, Francesco Checchi

**Affiliations:** 1 Department of Ecology and Evolutionary Biology, Princeton University, Princeton, New Jersey, United States of America; 2 London School of Hygiene & Tropical Medicine, London, United Kingdom; 3 School of Public Health, The University of Hong Kong, Hong Kong, People's Republic of China; 4 PRESTO, Japan Science of Technology Agency, Kawaguchi, Saitama, Japan; 5 Theoretical Epidemiology, Faculty of Veterinary Medicine, University of Utrecht, Utrecht, The Netherlands; University of Michigan and Howard Hughes Med. Inst., United States of America

## Abstract

Many infections can be transmitted between animals and humans. The epidemiological roles of different species can vary from important reservoirs to dead-end hosts. Here, we present a method to identify transmission cycles in different combinations of species from field data. We used this method to synthesise epidemiological and ecological data from Bipindi, Cameroon, a historical focus of gambiense Human African Trypanosomiasis (HAT, sleeping sickness), a disease that has often been considered to be maintained mainly by humans. We estimated the basic reproduction number 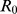 of gambiense HAT in Bipindi and evaluated the potential for transmission in the absence of human cases. We found that under the assumption of random mixing between vectors and hosts, gambiense HAT could not be maintained in this focus without the contribution of animals. This result remains robust under extensive sensitivity analysis. When using the distributions of species among habitats to estimate the amount of mixing between those species, we found indications for an independent transmission cycle in wild animals. Stochastic simulation of the system confirmed that unless vectors moved between species very rarely, reintroduction would usually occur shortly after elimination of the infection from human populations. This suggests that elimination strategies may have to be reconsidered as targeting human cases alone would be insufficient for control, and reintroduction from animal reservoirs would remain a threat. Our approach is broadly applicable and could reveal animal reservoirs critical to the control of other infectious diseases.

## Author Summary

Gambiense sleeping sickness is a disease transmitted by tsetse flies that mostly affects rural populations in sub-Saharan Africa. Although the parasite that causes the disease can be found in many different wild and domestic animal species, the disease has often been claimed to be maintained mostly by humans. Currently, fewer than 10,000 human cases are reported per year across Africa, and it has been suggested that elimination of gambiense sleeping sickness is feasible. We analysed human and animal case data from a well-known endemic focus of sleeping sickness in Cameroon, to quantify the contribution of the different species to the circulation of the parasite. In a wide range of scenarios, we found that animals are crucial for maintenance in the disease. When informing our model by the distribution of species among habitats as measured in the field, we found indications for independent transmission cycles in animals. This suggests that a risk of reintroduction from animal into human populations would remain even if the disease were eliminated from those human populations.

## Introduction

Many infections can be transmitted between animals and humans [1]. Human African Trypanosomiasis (HAT, sleeping sickness) is a vector-borne disease caused by parasites of the species *Trypanosoma brucei* and transmitted by flies of the genus *Glossina* (tsetse flies) [2]–[7]. While the east African form of HAT, caused by *T. brucei rhodesiense*, is a zoonosis with a well-described animal cycle in cattle and wild species, the more chronic west African form, caused by *T. brucei gambiense*, is often considered a human disease and causes more than 95% of reported cases in humans [8]. Gambiense HAT is endemic in 24 countries and deadly if untreated.

While *T. b. gambiense* has been found in numerous domestic and wild species [2]–[5], [9]–[13] and transmission between humans and other species been shown to occur both experimentally [9] and naturally [14], the exact role of animals in gambiense HAT epidemiology remains an unsolved puzzle [15], [16]. Are they sporadic dead-end hosts, or could they be an important factor for maintaining transmission?

Generally, the incidence of gambiense HAT can be brought to very low levels just by treating human cases, and indeed the latter strategy alone appeared to be sufficient for eliminating gambiense HAT from the island of Bioko in Equatorial Guinea [17]. Such observations have given rise to the notion that *T. b. gambiense* does not spread in animal populations without the presence of humans. However, the parasite was recently detected in flies on Bioko [18], suggesting that there is ongoing circulation of the parasite, with the existence of a wild animal reservoir appearing plausible given the lack of detected cases in humans or domestic animals on Bioko. The existence of self-sustained cycles of infection in animals could jeopardise efforts towards gambiense HAT elimination.

One of the very few systematic efforts to link the presence of *T. b. gambiense* in different animal species to human cases was a survey performed over several years in the historical focus of Bipindi, Cameroon, in response to the detection of 44 cases in humans by a newly-installed surveillance network in 1998/99 [19]. Subsequently, data on *T. b. gambiense* prevalence in domestic [13] and wild animal species [12], as well as in tsetse flies [20], biting preferences [21] and the distribution of species among different types of habitat [22] were collected, providing a rich epidemiological and ecological dataset. Synthesising these data in a common modelling framework presents a mathematical and conceptual challenge.

Here, we use the concept of the next-generation matrix (NGM) [23] to understand the transmission dynamics of gambiense HAT in Bipindi. The NGM describes the number of secondary cases caused in each species by an infected host or vector of any (other or the same) species and allows the generalisation of a classical epidemiological quantity, the basic reproduction number 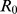, to a situation in which there are different types of hosts or host species. Defining 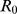 as the spectral radius 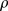 or largest eigenvalue of the NGM generalises the endemic threshold properties of 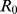 in single-host systems, in the sense that if 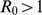 there can be sustained transmission and if 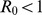 there cannot. We use a mathematical model of gambiense HAT transmission to understand the prevalence observed in hosts and vectors and estimate the elements of the NGM.

Mathematical models of gambiense HAT transmission involving humans only [24] or humans and one animal species [25]–[27] have been derived previously and have yielded valuable insights into HAT epidemiology. For example, it has been shown that there are scenarios in which HAT may require a non-human reservoir host for persistence [25]. From sensitivity analysis of the parameters entering 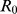 it has been concluded that the proportion of bloodmeals the vector takes from humans is the most important factor, indicating that variation in the exposure to tsetse flies could explain the spatial distribution of *T.b. gambiense* [26]. Sensitivity of those parameters to expected climate change (albeit for *T.b. rhodesiense*) suggests a shift in the geographical range of infection risk [27]. All these results and, more generally, estimates of 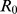 for gambiense HAT have not been based on data collected from animals, vector and human systems within the same focus, and instead have relied on the combination of parameter values estimated or drawn from different literature sources. The method we present here is broadly applicable to vector-borne diseases with a potential animal reservoir, and is designed to be informed by data from field surveys. It is based on the premise that the system is in endemic equilibrium, an assumption we revisit in the Discussion section. We show that, in an equilibrium scenario, both 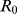 and the contribution of different species or groups of species can be estimated using only data on (a) relative prevalence of infection in different host species and (b) the distribution of bites of the vector on different species. We use this method to assess the potential of each species or combinations of species to maintain gambiense HAT transmission in Bipindi. Further, we extend our method to incorporate ecological data (the distribution of species across different habitats) and use this to perform extensive sensitivity analysis.

## Methods

The analysis is based on the assumption that the system has been observed in an equilibrium state. This allows us to calculate the forces of infection in all species from measured prevalences. Using these, we derive the next-generation matrix (NGM) in all host and vector species participating in the transmission cycle. Assuming that the system is in an endemic equilibrium, implies that 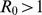 (see linear stability analysis in Supporting Text S1).

### Data sources

The human case data come from two active screening campaigns, performed in November 1998 and February 1999, following the discovery of infected blood sera from the Bipindi area, previously practically ignored in medical surveys [19]. The first of these campaigns concentrated on two neighbouring villages and found 26 infected cases. The second one expanded to a total of 15 villages (including the two villages screened in the first survey), detecting 18 further cases, of which 16 were found in the two villages visited during the first campaign. The data from domestic animals come from a survey performed in 5 villages of the Bipindi area in 2003/04 [13], including the two villages containing most of the human cases. The data from wild animals come from surveys performed in Bipindi between 1999 and 2001 [12]. The case data are summarised in Tables 1 and 2.

**Table 1:** Summary of sampling campaigns.

Date	Survey	sampled	positive
Nov 1998	Humans (2 villages)	1269	26
Feb 1999	Humans (15 villages)	3519	18
1999–2001	Wild animals	832	18
2003/04	Domestic animals	875	27

Number of sampled humans and animals, and number positive for *T. b. gambiense*. 500 of the 832 wild animals sampled were from the Bipindi area (15 positive). 204 of the 875 domestic animals were from the Bipindi area (8 positive).

**Table 2:** Summary of case data: + indicates positive for *T. b. gambiense*, and 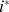 the resulting equilibrium prevalence.

Name	Scientific name	Samples	+	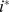	Source
Human	*Homo sapiens*	3641	44	0.012	[19]
Sheep	exact species unknown	267	18	0.067	[13]
Goat	exact species unknown	264	8	0.030	[13]
Pig	exact species unknown	307	1	0.0033	[13]
White-eyelid mangabey	*Cercocebus torquatus*	5	1	0.20	[12]
Greater white-nosed monkey	*Cercopithecus nictitans*	80	4	0.050	[12]
Blackstriped duiker	*Cephalophus dorsalis*	16	1	0.062	[12]
Blue duiker	*Cephalophus monticola*	200	4	0.020	[12]
Brush-tailed porcupine	*Atherurus africanus*	100	2	0.020	[12]
Giant rat	*Cricetomys gambianus*	125	3	0.024	[12]
Small-spotted genet	*Genetta servalina*	8	1	0.13	[12]
Two-spotted palm civet	*Nandinia binotata*	29	2	0.069	[12]

For our analysis, since we were interested in the potential for animal reservoirs to maintain gambiense HAT, we attempted to make our estimates conservative in that regard. We included all the villages screened in Bipindi for our basic estimate of prevalence in humans, as the area comprising these villages region compares well to where the tested animals came from (see the Results section for sensitivity analysis on the human prevalence estimate). Moreover, we combined the two surveys in human populations into a single prevalence estimate, which is equivalent to assuming that the two surveys took place at the same time and ensures we do not underestimate prevalence due to medical interventions in response to the first survey (i.e., to estimate prevalence we took all infected cases found in both screening surveys as enumerator and the combined population of the villages screened as denominator). The data from both domestic and wild animals were collected later, and are very likely to be affected by vector control installed after the human cases were detected, which could be expected lower the prevalence in all species. Since we did not have access to animal case data separated by location and species, we used all the survey data. As a consequence, in both the data from domestic and wild animals, the prevalence we are using is lower than the one reported from Bipindi alone (all species combined). In summary, we are likely to underestimate equilibrium prevalence in animals, in line with our attempt to be conservative in that regard.

In the analyses presented below we assumed infection among a given species to be binomially distributed with fixed infection probability corresponding to an average equilibrium prevalence. The likelihood 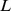 for equilibrium prevalence 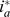 in species 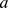 (equivalent to the probability of being infected), given 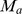 cases detected among 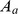 sampled animals, is then proportional to a beta distribution,
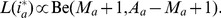
(1)This quantifies the uncertainty resulting from small sampling sizes (the smallest being White-eyelid mangabeys with only 5 sampled animals), with correspondingly wide confidence intervals.

All other parameters are drawn from flat distributions using Latin Hypercube Sampling [28], with ranges given in Supporting Text S2.

### Model assumptions

In setting up the model, we made the following biological assumptions:

- Population sizes are constant with no demographic stochasticity.
- The duration of the first stage of the disease (equivalent to the duration of infectiousness in our model) is exponentially distributed, as the evidence suggests [29]. Moreover, we assume that there is no long-term chronic carriage, although there is some evidence of that they sometimes occur [29].
- We do not have to distinguish between teneral and non-teneral flies. Generally, the susceptibility of a tsetse fly to midgut infection with trypanosomes decreases if they are not infected after the first bloodmeal. We found no qualitative difference when considering a model in which only teneral flies (i.e., the ones that have not had their first blood meal) can be infected (see Supporting Text S1). Moreover, the probability of infection we estimate for flies (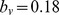) is consistent with what one would expect as average probability of infection of tsetse flies [30].
- The transmission rate of an infected host or vector does not change over time. This is consistent with findings that transmissibility of trypanosomes is independent of parasitemia [31].
- Biting preference is as measured by Polymerase Chain Reaction (PCR) on blood in flies that have fed. This implies that blood specimens were randomly sampled and that the test is equally sensible to all bitten species.

### Basic model

Assuming random mixing and uncorrelated bites, a simple transmission model for gambiense HAT transmission between 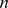 host and one vector species is given by the system of 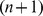 ordinary differential equations, based on the Susceptible-Infected-Susceptible (SIS) model
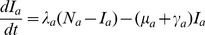
(2a)

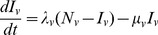
(2b)where 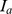 is the number of infected of host species 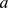, 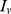 is the number of infected vectors, 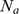 and 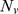 are the total population sizes of host species 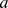 and vectors, respectively, 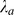 and 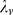 are the forces of infection acting on host species 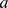 and the vector, respectively, 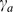 is the rate at which infected hosts of species 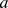 lose infectiousness (through recovery or death), and 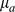 and 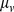 are the natural death rates (and birth rates, assuming constant population sizes) of host species 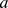 and the vector, respectively.

### Forces of infection

The forces of infection are
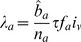
(3a)

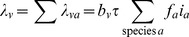
(3b)where 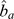 is the probability for an infectious bite on a susceptible host of species 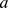 to lead to infection, rescaled by the ratio of vector to host population sizes, 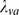 is the force of infection exerted by species 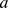 on vectors, 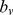 is the probability that an infectious bite by a susceptible vector leads to transmission of the parasite and establishment in the vector midgut. These transmission probabilities are treated as unknown quantities to be estimated. The other parameters are measured quantities: 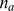 is the relative population density of species 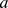 compared to all other hosts, 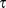 is the biting rate of vectors, 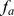 is the fraction of bites taken on species 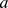, and 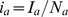 and 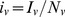 are the prevalence of infection in species 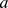 and vectors, respectively.

Assuming that the system is in equilibrium, we get a relation between force of infection and prevalence,
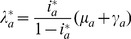
(4a)

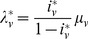
(4b)where the asterisk denotes equilibrium quantities.

### Next-generation matrix

The NGM describes transmission between different vector and host species by mapping the distribution of primary cases to the distribution of secondary cases [23]. Once fully quantified, the matrix allows to identify host species that can maintain transmission of a given infection [32]. That is, we can distinguish between maintenance and non-maintenance hosts by calculating the host-specific reproduction number 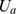 of (group of or single) host species 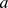, which is interpreted as the average number of secondary cases per generation caused (via the vector) by a single primary case belonging to 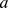 in the absence of hosts other than 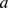. If 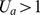, host(s) 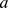 can maintain gambiense HAT transmission on its (their) own. This formalises the definition of maintenance hosts given in [33].

### Correlated bites

To capture the impact of correlated bites on model dynamics, we separate our vector class 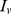 into 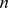 classes and denote these 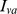, the number of infected vectors that have last fed on host species 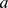 If 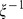 is the average time spent feeding on a given species, the dynamical equations for 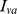 are
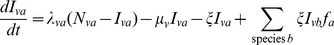
(5)where 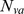 is the total number of vectors that have last fed on species 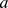. In equilibrium, this can be solved for which is used to parametrise the NGM and can be extended to groups of species (see Supporting Text S1).

### Habitat separation

Extending the scenario of correlated bites to known differences in habitat, we introduce a mixing matrix 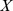, the elements 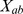 of which describes how likely a vector is to switch (and potentially transmit infection) from species (or group of species) 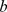 to species (or group of species) 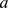. The dynamical equations for 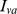 then become
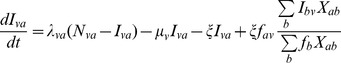
(6)which, again, is used to parametrise the NGM.

With the densities 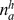 (or presence/absence) of the different species 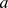 in different habitats 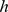 are given, we estimated mixing rates 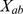 to
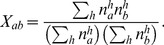
(7)


### Numerical methods

Simulations were performed using the Gillespie algorithm [34]. All parameter estimations where there was no analytical solution were performed using Powell's hybrid method [35] as implemented in the GNU Scientific Library [36].

## Results

We first state the general result relating the basic reproduction number 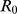 and host- and group-specific reproduction numbers 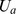 to endemic prevalences and biting preferences, before applying this to the scenario of gambiense HAT transmission in Bipindi.

### Identifying transmission cycles

In a multi-host system, the basic reproduction number 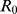 is defined as the spectral radius of the NGM. In the Supporting Text S1, we show that when we are dealing with only one vector species the basic reproduction number is
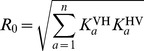
(8)where the sum is over all host species 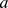 and 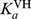 is the average number of infected vectors caused in a completely susceptible vector population by a single host of species 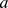, and 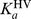 as the average number of infected hosts of species 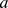 caused by a single vector in a completely susceptible host population. A special case of this equation for a system composed of humans and one animal species has previously been derived in [26]. The host-specific reproduction number [32] of a group 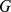 of host species, or their contribution to the basic reproduction number 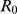, is
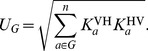
(9)This is equivalent to the value 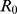 would take in a system of only the subset of species in 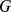. The summands are related to the forces of infection via
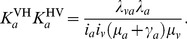
(10)In equilibrium, we can use Eqs. (3) and (4) to rewrite this as
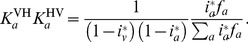
(11)We can use this to calculate the basic reproduction number given only equilibrium prevalence in the vector (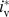) and all host species (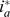) and vector biting preference 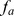 (the fraction of bites taken on species 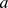),
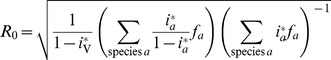
(12)This does not require any information on vector biting behaviour, host or vector population sizes, or within-host infection dynamics.

### Animal reservoirs of gambiense HAT in Bipindi

**Figure 1:**
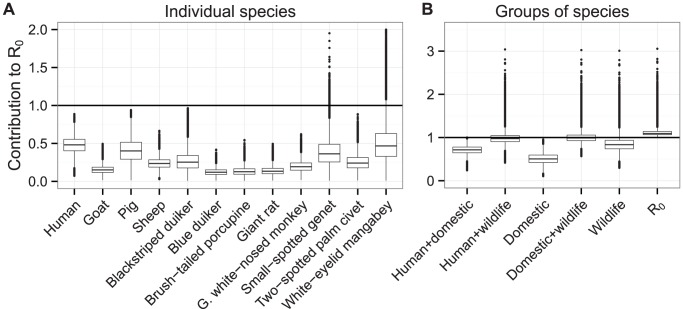
Contributions of species and species groups to 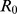 under random mixing. (a) The contributions of different species to 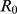 under the assumption of random mixing between vectors and hosts. (b) The contribution of different sets of species to 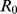 under the assumption of random mixing between vectors and hosts. In both plots, the y-axis shows the values of 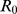 which would be found in a system of only the given (set of) species and vectors, the central line indicating the most likely value, upper and lower edges the interquartile range, the outer lines 1.5 times the interquartile range, and individual dots outlier results. The rightmost data point in (b) shows the estimate for 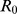 in the whole system (all species combined). Outliers for white-eyelid mangabeys with 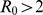 (0.1% of values) are not shown.

For the focus we investigated, in the baseline scenario of random mixing of vectors with the different host species (proportional to biting preference as measured) we found that the median value of 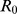 was 1.1 (95% CI 1.0, 1.3) (Fig. 1). The contribution of humans (i.e., the hypothetical value of 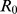 in a system of only humans and vectors) was 0.5 (0.2, 0.7). When testing for potential cycles of sustained transmission in groups of species, we found that 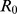 in domestic animals was 0.5 (0.3, 0.8). When adding humans to the system, 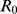 increased to 0.7 (0.5, 0.9). In wild animals, 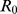 was 0.8 (0.6, 1.2), with a likelihood of 0.14 of being greater than 1. In all animals (wild and domestic), 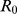 was 1.0 (0.8, 1.3), with a likelihood of 0.46 of being greater than 1.

These results are in contrast to the notion of gambiense HAT as human disease with only accidental animal hosts [7]. However, we could be underestimating the prevalence in (and, consequently, the importance of) humans for two main reasons: (i) active case detection campaigns might not have detected all cases in the population screened due to problems with diagnostic sensitivity [37], [38] or the presence of asymptomatic carriers with low parasitemia [29] (note that our denominator is the population screened, so screening attendance does not change our estimate as long as individuals screened are chosen randomly), and (ii) the denominator at risk might in fact not be the entire population screened if the risk of infection is unevenly distributed. The effects of these two are equivalent and multiplicative: If a fraction 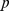 of cases are detected, and a fraction 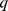 of the population is involved in the transmission cycle, the measured prevalence is 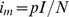 and true prevalence is 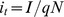, such that 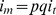.

**Figure 2:**
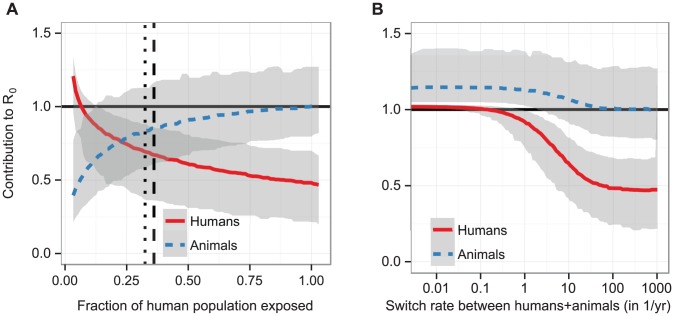
Human and animal contributions to 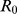 under different model scenarios. (a) The contribution of the human (red, solid) and animal (blue, dashed) populations to 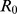 as a function of the fraction of the population exposed to bites of the vector, shown here as effective population size 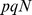. The vertical dashed line indicates the fraction of the population in the main endemic area [19], and the dotted line 90% of that population, a low estimate for screening efficacy [37]. (b) The contribution of the human (red, solid) and animal (blue, dashed) populations to 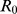 as a function of the rate of host switching between a species, given in units of (number of switches)/year/fly. In both plots, the y-axis shows the values of 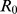 which would be found in a system of only humans and the vector. The lines show the best estimate, and the light grey areas contain the smoothed (2.5%, 97.5%) quantile range, obtained from the binomial likelihood profiles and Latin hypercube sampling of parameter ranges (see Supporting Text S2).

If we increase the prevalence in humans to account for these potential sources of bias, 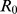 of the system with only animals and vectors decreases (Fig. 2a). More specifically, if only the 40% of the population of Bipindi living in the two villages with most of the detected cases [19] are at risk of infection, and if we incorporate a low estimate of 90% for screening sensitivity [37], the likelihood for 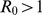 in animals decreases to 0.13, but the likelihood for 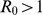 in humans is still less than 0.01. Only if we further reduce the population at risk to less than 20% of these villages does the likelihood for 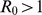 in animals drop to less than 0.01. In that case, the likelihood for 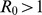 in humans is 0.59.

A second source of potential bias could arise if subsequent bites of the same fly were correlated, or if a fly taking a blood meal on a given species or group of species had a higher probability of biting a host of the same species or another species in that group again [39], [40]. Our analysis attributes human infection either to other human infections (via a vector) or to spillover from animal reservoirs (again via a vector). If the two kinds of host population are fully epidemiologically linked (i.e., if we assume random mixing), then the analysis inevitably attributes many of the cases in the population with lower (weighted) prevalence to spillover from the population with higher (weighted) prevalence. The less linkage there is the less likely this is to happen, and eventually 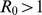 in the low-prevalence population is required to explain persistence. When we considered a system of two transmission cycles, one containing humans and domestic animals and the other one wild animals (i.e., a system in which there is a sylvatic cycle separate from the human/domestic animal cycle), the human contribution to the system was not enough to guarantee 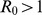 in the system of humans and domestic animals. When humans were considered to be part of a transmission cycle completely separate from animals, we got 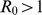 in both the human and the (wild and domestic) animal cycle. Introducing only occasional transfer of infection between species, however, means the observed data are not compatible with sustained transmission in the human-vector cycle, with a threshold appearing at a rate of switching of about 1/year (Fig. 2b). 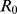 in humans was greater than 1 with likelihood greater than 0.01 only when vectors switched between species less than once per year. Comparing these with an average fly life expectancy of about one month, this would mean that most flies never change host species in their lifetime, an unrealistic scenario given that in practice flies cannot afford to restrict themselves to one host type. Independent transmission cycles in animal reservoirs, on the other hand, have a likelihood greater than 0.5 for any rate of switching less than 30/year, corresponding to 2–3 host switches per fly in its lifetime.

**Figure 3:**
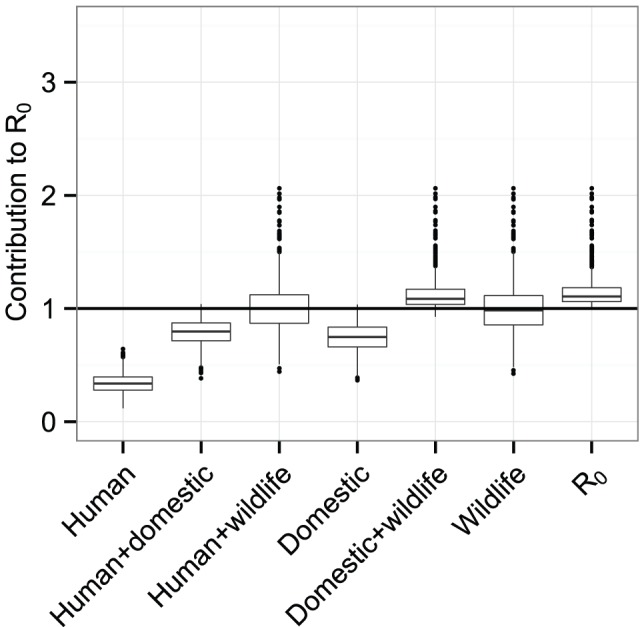
Contributions of species groups to 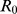 under habitat-specific mixing. The contributions of different groups of species to 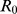 under the assumption of mixing proportional to habitat overlap of hosts. Hosts are grouped according to the habitats they can be found in, with random mixing within these groups and switching occurring at a third of the biting rate between the groups. The y-axis shows the values of 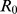 which would be found in a system of only the given set of species and vectors, the central line indicating the most likely value, upper and lower edges the interquartile range, the outer lines 1.5 times the interquartile range, and individual dots outlier results. The rightmost data point in shows the estimate for 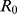 in the whole system (all species combined).

To inform this analysis with ecological measurements of habitat distributions of the species found to host gambiense HAT in Bipindi [22], we incorporated the overlap of habitat ranges between animals in our derivation of the NGM. This version of the model does not support a human-only transmission cycle, and suggests that a sylvatic cycle is possible. Separating the different species by the habitats they can be found in yielded likelihood 0.48 for 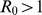 in wildlife species only (Fig. 3), and likelihood 0.97 for 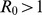 in all animal species if switches between groups of species happened at a third of the biting rate.

**Figure 4:**
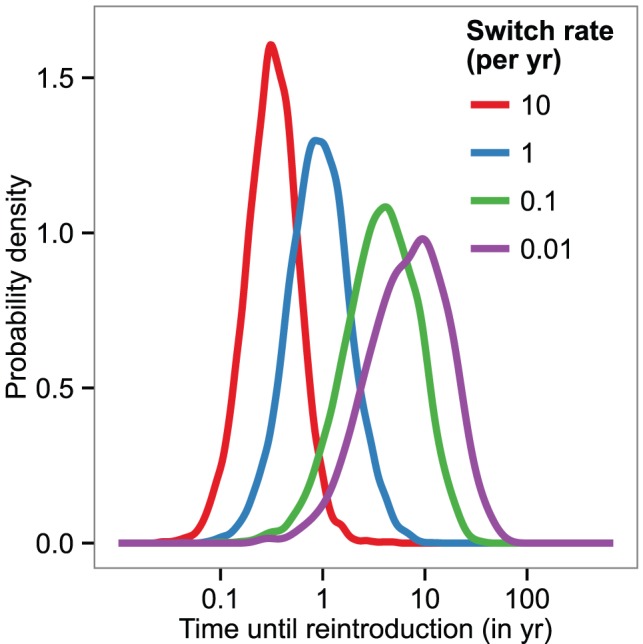
Reintroduction periods after elimination from the human and domestic population. The probability distribution of reintroduction periods for different rates of host switching (given in units of (number of switches)/fly/year) between a human/domestic and a wild animal subsystem (with random mixing within each of these two subsystems), given in years. The values were obtained from 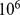 stochastic simulations, initialised with the prevalence in animal populations as measured in Bipindi, but with no infection present in humans, domestic animals, or human-associated vectors. Simulations were initialised with 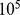 vectors, based on the number of around 2,000–3,000 flies captured in the area through entomological surveys lasting a few days [45]. We considered reintroduction to have occurred once there were 2 cases in humans at any given time.

We performed simulations of the different model variants, with a particular focus on how long it would take for the disease to become re-established in a human population from which it had previously been eliminated. We tested different rates of vector switching between a human/domestic and a wild animal cycle, as well as other configurations of cycles. As the rate of switching decreased, the time it can take for cases to reappear in the human population increased (Fig. 4). For rates of switching greater than 1/year, reintroduction usually occured within a year or less. When, on the other hand, switches between humans, domestic animals and wild animals were as rare as 0.01/year per fly (i.e., only one in 1000 flies ever switched between these subsystems) it could take 10 years or longer for infection to be transferred between them.

## Discussion

We have developed a mathematical model to assess transmission dynamics in a focus of gambiense HAT, and analysed it incorporating a variety of epidemiological and ecological measurements, providing one of the first estimates of 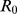 in gambiense HAT from field data. If vectors and hosts mix randomly, we only need the prevalence in the different vector and host species, as well as the distribution of bites on host species, to determine the NGM and 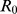. In this case, the available data strongly suggest that *T. b. gambiense* cannot be sustained in a human (and vector) population alone, whereas independent transmission cycles in animal reservoirs are possible in a realistic parameter range. When reducing the human population at risk, we could not rule out the possibility of transmission cycles in humans and vectors. However, these occured only with a very small likelihood corresponding to very specific parameter combinations unless it was only a very small fraction of the human population that was exposed to the potential infection. While there are occupational hazards associated with trypanosomiasis infection (especially hunting [41]), these do not seem enough to explain such strong heterogeneity in risk.

When we relaxed the assumption of random mixing to reduce the amount of infection transfer between humans and other species, human transmission cycles were only possible in parameter regimes where there was a parallel transmission cycle in wildlife. When we informed this analysis with measured distributions of species among habitats, independent transmission cycles in animals occured with high probability. Simulating the transmission dynamics of the model with different rates of vector switching between three subsystems of humans, domestic animals and wild animals, we observed that unless switching was rare, reintroduction of infection in humans usually occurred within less than a year. When, on the other hand, such a switch happened only in a minority of vector lifetimes, reintroduction could take many years, and there was the possibility a human-only cycle in parallel with a separate sylvatic cycle. The disease-free periods of 10 or more years subsequent to human case control that have been observed [17] would point to such a scenario. However, the effect of vector control combined with delayed recognition of new outbreaks due to infrequent screening and lack of gambiense HAT testing in routine health services may also explain long delays observed between apparent elimination of *T. b. gambiense* from a focus and its re-activation.

Our analysis hinges on the assumption of equilibrium, which allowed us to estimate the force of infection from observed prevalence. While fluctuations in the density of the different species or the incidence of infection that they experience are likely, the slow dynamics of gambiense HAT combined with the long history of endemic transmission in Bipindi [42] would appear to justify the assumption of stationarity. Still, since the data underlying our study were taken at different points throughout the year, strong seasonality could mean that the measurements were not a good reflection of the average state of the system, as well as raising theoretical issues in linking persistence of an endemic disease to the value of 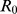 [43]. While we cannot resolve this issue on the basis of the available data, we note that vector density was found not to vary significantly in the study area [44], and that the progression of gambiense HAT is slow relative to the progression of seasons, so that fluctuations in tsetse fly density need not translate into significant changes in prevalence. Further, it is worth noting that more detailed data on incidence would enable relaxation of the model assumptions and direct estimation of the force of infection. Moreover, molecular typing of parasite material could be used to quantify the contribution of non-human hosts to the force of infection in humans.

Clarifying the precise role of animal hosts in maintaining transmission has important implications for elimination strategies. If wild animals can maintain *T. b. gambiense* in a separate transmission cycle, elimination (the permanent interruption of transmission) will be difficult to achieve with a strategy based on human case detection alone. At the same time, all our estimated likely values 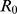 are very close to 1, suggesting that the disease should be controllable, especially if vector control is introduced and maintained. Beyond maintenance, animals could play a role in transmitting infections between communities within a given focus or indeed (re-)introduction into old, extinct foci or new areas. Gambiense HAT has remained a west and central African disease confined to persistent foci in spite of large-scale population movements around the continent. If transmission could be maintained in a human-vector system alone, one would expect the distribution of the disease to be more diffuse. Instead, one could speculate that restrictions of animal host ranges are at least to some degree responsible for the observed distribution. An intriguing hypothesis that arises from our results is that the apparent decline in gambiense HAT burden in many areas of west Africa (e.g., Gambia, Sierra Leone, Liberia, Nigeria) where it was previously highly endemic might be attributable mainly to the reduction in wildlife habitats and populations in these regions over the past decades.

We have concentrated on an gambiense HAT focus in a region with a well-documented history of endemic transmission [42]. Extrapolation of our results to other settings warrants caution. Focus-specific levels of parasite strain virulence, vector competence or human susceptibility could combine to ensure sustained transmission in human-vector systems elsewhere. Similarly, species and distributions of domestic and wild animals vary considerably across foci. Nevertheless, this study offers an attractive explanation for the mysterious disappearance and re-activation of gambiense HAT foci throughout Africa. Our method is easily generalised to other foci, and further studies on the ecology and epidemiology of *T. b. gambiense* across different areas would firmly establish the role of wild and domestic animals in the maintenance of sleeping sickness, and to systematically assess the prospects of elimination efforts.

In this study, we analysed one of the largest systems for which the NGM has been quantified from field data. Combined with efforts to measure infection prevalence in both humans and animals, our model framework could be applied to better characterise the role of animal hosts in the long-term control of many other diseases, such as yellow fever, rift valley fever or Chagas disease.

## Supporting Information

Text S1 **Model formulation.** Contains details of relating the NGM to the basic and host-specific reproduction numbers in a multi-host, mulit-vector system; a detailed description of the modelling framework for HAT; and a brief overview of possible model extensions.
(PDF)

Text S2 **Data.** Contains the gambiense HAT prevalence data in different host and vector species as well as measured biting preferences and other parameters of the gambiense HAT transmission model.
(PDF)
