# Supplementary material for: Identifying Transmission Cycles at the Human-Animal Interface: The Role of Animal Reservoirs in Maintaining Gambiense Human African Trypanosomiasis
Source: PLoS Comput Biol. 2013 Jan 17;9(1):e1002855. doi: 10.1371/journal.pcbi.1002855 (PMC3547827; doi:10.1371/journal.pcbi.1002855)
Supplement: Text S1 — Model formulation. Contains details of relating the NGM to the basic and host-specific reproduction numbers in a multi-host, mulit-vector system; a detailed description of the modelling framework for HAT; and a brief overview of possible model extensions. (PDF) [file pcbi.1002855.s001.pdf]

Supplementary Text S1: Model formulation

Identifying transmission cycles at the human-animal interface:
The role of animal reservoirs in maintaining
gambiense Human African Trypanosomiasis

Sebastian Funk, Hiroshi Nishiura, Hans Heesterbeek,
W. John Edmunds, Francesco Checchi

Contents

1 General setup **2**
1.1 The next-generation matrix 2
1.2 Determining independent transmission cycles 3

2 Model scenarios for HAT **5**
2.1 Random mixing 5
2.2 Correlated bites 9
2.3 Interaction rates from habitat overlap 12

3 Model extensions **13**
3.1 Incubation period 14
3.2 Teneral flies 14

References **15**

1 General setup

We are dealing with n host species labelled $a = 1 \dots n$, and m vector species labelled $v = 1 \dots m$. We are trying to evaluate which species or combinations of species can maintain infection (Haydon et al., 2002).

We will employ the following notation conventions: matrices will be denoted by bold capital latin letters (e.g., $\mathbf{K}$), counts by normal capital letters (e.g., N), rates by small greek letters (e.g., ξ), probabilities and proportions by small latin letters (e.g., i). Measurable rates or probabilities will be typed in bold (e.g., $\boldsymbol{\mu}$) and ones estimated or unkown in normal font (e.g., b_v). Equilibrium quantities will be denoted with an asterisk (e.g., i^*). Host species will be labelled $a, b, \dots$ and vector species $v, w, \dots$ Eigenvalues will be labelled λ (not to be confused with forces of infection on hosts λ_a and vectors λ_v).

1.1 The next-generation matrix

The next-generation matrix (NGM) is a quantity that provides a link between the number of new infected in each generation of infection in different host (or vector) types or species. The elements of the NGM denote the average number of infections caused by a single infected host (vector) in a completely susceptible population of the same or another host (vector) species. Following Diekmann et al. (2010), we can calculate the NGM after decomposing the Jacobian matrix of the system into a transmission part T and a transition part C

$$\mathbf{K} = -\mathbf{T}\mathbf{C}^{-1} \tag{1}$$

In the case of a vector-borne disease and in the absence of vertical transmission, the transmission matrix T is of the form

$$\mathbf{T} = \begin{pmatrix} 0 & \mathbf{T}^{\mathrm{VH}} \\ \mathbf{T}^{\mathrm{HV}} & 0 \end{pmatrix} \tag{2}$$

where $\mathbf{T}^{\mathrm{VH}}$ is an $m \times n$ matrix of transmission terms from the host to the vector and $\mathbf{T}^{\mathrm{HV}}$ is an $n \times m$ matrix of transmission terms from the vector to the host. The transition matrix $\boldsymbol{C}$ is of the form

$$\mathbf{C} = \begin{pmatrix} \mathbf{C}^{\mathrm{V}} & 0 \\ 0 & \mathbf{C}^{\mathrm{H}} \end{pmatrix} \tag{3}$$

where $\mathbf{C}^{\mathrm{V}}$ is a matrix of terms describing mortality of the vector species and $\mathbf{C}^{\mathrm{H}}$ is a matrix of terms describing recovery and mortality of the different host species. Since the inverse of a block diagonal matrix is a block diagonal

matrix of the inverses of the blocks, the NGM is of the form

$$\begin{aligned}\mathbf{K} &= -\mathbf{T}\mathbf{C}^{-1} \\ &= \begin{pmatrix} 0 & \mathbf{T}^{\mathrm{VH}} \\ \mathbf{T}^{\mathrm{HV}} & 0 \end{pmatrix} \begin{pmatrix} \left(\mathbf{C}^{\mathrm{V}}\right)^{-1} & 0 \\ 0 & \left(\mathbf{C}^{\mathrm{H}}\right)^{-1} \end{pmatrix} \\ &= \begin{pmatrix} 0 & \mathbf{T}^{\mathrm{VH}}\left(\mathbf{C}^{\mathrm{H}}\right)^{-1} \\ \mathbf{T}^{\mathrm{HV}}\left(\mathbf{C}^{\mathrm{V}}\right)^{-1} & 0 \end{pmatrix}\end{aligned} \tag{4}$$

If there is no vertical transmission, the NGM $\mathbf{K}$ for a vector-borne disease will only ever contain two blocks of elements denoting transmission between the host and vector.

$$\mathbf{K} = \begin{pmatrix} 0 & \mathbf{K}^{\mathrm{VH}} \\ \mathbf{K}^{\mathrm{HV}} & 0 \end{pmatrix} \tag{5}$$

where $\mathbf{K}^{\mathrm{VH}}$ is a (sub-)matrix containing only elements for host-to-vector transmission, and $\mathbf{K}^{\mathrm{HV}}$ contains only elements for vector-to-host transmission. The eigenvalues λ of that matrix can be found by solving

$$\begin{aligned}0 &= \det(\mathbf{K} - \lambda \mathbb{1}^{(n+m)}) \\ &= (-\lambda)^{n-m} \det\left(-\lambda^2 \mathbb{1}^m + \mathbf{K}^{\mathrm{VH}}\mathbf{K}^{\mathrm{HV}}\right)\end{aligned} \tag{6}$$

where $\mathbb{1}^x$ is the x-dimensional unity matrix. In other words, the non-zero eigenvalues of $\mathbf{K}$ are the square roots of the eigenvalues of the $m \times m$ matrix $\mathbf{K}^{\mathrm{VH}}\mathbf{K}^{\mathrm{HV}}$.

1.2 Determining independent transmission cycles

In a multi-host system, the basic reproduction number R_0 is defined as the spectral radius of $\mathbf{K}$, and for a vector-borne disease without vertical transmission it is given by

$$R_0 = \rho(K) = \sqrt{\rho(\mathbf{K}^{\mathrm{VH}}\mathbf{K}^{\mathrm{HV}})} \tag{7}$$

It represents a threshold in that an infection can establish itself in a susceptible population if $R_0 > 1$. Usually, this is also the condition for persistence (see Linear Stability Analysis below).

To determine reservoirs, we calculate the host-specific (U) and host-excluded (Q) reproduction numbers (Roberts and Heesterbeek, 2003; Nishiura et al., 2009)

$$U_A^{\mathrm{V}} = \rho((\mathbf{P}_{\mathrm{V}} + \mathbf{P}_A)\mathbf{K}) \tag{8}$$

and

$$Q_A^{\mathrm{V}} = \rho((\mathbf{P}_{\mathrm{V}} + \mathbb{1}_{\mathrm{V}} - \mathbf{P}_A)\mathbf{K}) \tag{9}$$

where $\rho(\boldsymbol{A})$ is the spectral radius of matrix $\boldsymbol{A}$, $\mathbf{P}_A$ is some projection matrix selecting for a set of (one or more) species that we are interested in, and the vector projection $\mathbf{P}^{\mathrm{V}}$ and vector-excluded unity matrix $\mathbb{1}_{\mathrm{V}}$ are defined as

$$\mathbf{P}^{\mathrm{V}} = \begin{pmatrix} \mathbb{1}^m & 0 & \cdots & 0 \\ 0 & 0 & \vdots & \vdots \\ \vdots & \vdots & \ddots & 0 \\ 0 & \cdots & \cdots & 0 \end{pmatrix} \qquad \text{and} \qquad \mathbb{1}_{\mathrm{V}} = \mathbb{1}^{m+n} - \mathbf{P}^{\mathrm{V}} \tag{10}$$

A species a is a maintenance host species if $U_a^{\mathrm{V}} > 1$ and a reservoir species if, in addition, $Q_a^{\mathrm{V}} < 1$. The analogue holds for groups of host species (Nishiura et al., 2009).

We now consider the case where there is only one vector species v. In that case, we have $m = 1$ and $\mathbf{K}^{\mathrm{VH}}\mathbf{K}^{\mathrm{HV}}$ is a 1x1 matrix. Writing K_a^{VH}, and K_a^{HV} for the elements K_{1a}^{VH} and K_{a1}^{HV} of the one-dimensional matrices $\mathbf{K}^{\mathrm{VH}}$ and $\mathbf{K}^{\mathrm{HV}}$, respectively, we get

$$\mathbf{K}^{\mathrm{VH}}\mathbf{K}^{\mathrm{HV}} = \sum_{a=1}^{n} K_a^{\mathrm{VH}} K_a^{\mathrm{HV}} \tag{11}$$

where K_a^{VH} can be interpreted as the average number of infected vectors caused in a completely susceptible vector population by a single host of species a, and K_a^{HV} as the average number of infected hosts of species a caused by a single vector in a completely susceptible host population. In this case, the basic reproduction number is

$$R_0 = \sqrt{\sum_{a=1}^{n} K_a^{\mathrm{VH}} K_a^{\mathrm{HV}}} \tag{12}$$

If we are interested in the role of species a, we get (writing a for the set $\{a\}$)

$$\begin{aligned} U_a^{\mathrm{V}} &= \rho((\mathbf{P}_{\mathrm{V}} + \mathbf{P}_a)\mathbf{K}) \\ &= \rho \begin{pmatrix} 0 & \cdots & K_a^{\mathrm{VH}} & \cdots & 0 \\ \vdots & \ddots & & & \vdots \\ K_a^{\mathrm{HV}} & & 0 & & \vdots \\ \vdots & & & \ddots & \vdots \\ 0 & \cdots & \cdots & \cdots & 0 \end{pmatrix} \\ &= \sqrt{K_a^{\mathrm{VH}} K_a^{\mathrm{HV}}} \end{aligned} \tag{13}$$

and

$$
\begin{aligned}
Q_a^{\mathrm{V}} &= \rho((\mathbf{P}_{\mathrm{V}} + \mathbb{1}_{\mathrm{V}} - \mathbf{P}_a)\mathbf{K}) \\
&= \rho \begin{pmatrix}
0 & K_1^{\mathrm{VH}} & \cdots & K_{a-1}^{\mathrm{VH}} & 0 & K_{a+1}^{\mathrm{VH}} & \cdots & K_n^{\mathrm{VH}} \\
K_1^{\mathrm{HV}} & 0 & \vdots & 0 & \vdots & 0 & \vdots & 0 \\
\vdots & \vdots & \ddots & \vdots & \vdots & \vdots & \vdots & \vdots \\
K_{a-1}^{\mathrm{HV}} & 0 & \cdots & \ddots & \cdots & \cdots & \cdots & \vdots \\
0 & \cdots & \cdots & \cdots & \ddots & \cdots & \cdots & \vdots \\
K_{a+1}^{\mathrm{HV}} & 0 & \cdots & \cdots & \cdots & \ddots & \cdots & \vdots \\
\vdots & \vdots & \vdots & \vdots & \vdots & \vdots & \ddots & \vdots \\
K_n^{\mathrm{HV}} & 0 & \cdots & \cdots & \cdots & \cdots & \cdots & 0
\end{pmatrix} \\
&= \sqrt{\sum_{b \neq a} K_b^{\mathrm{VH}} K_b^{\mathrm{HV}}}
\end{aligned}
\tag{14}
$$

2 Model scenarios for HAT

In the following, we will develop different versions of a simple transmission model of HAT, starting with the simplest version before discussion possible extensions.

2.1 Random mixing

In the simplest model, we assume that bites are uncorrelated and the probability of biting any host species is independent of which species was bitten previously by the same vector.

Dynamic system

The system is described by $(n + m)$ differential equations:

$$\frac{dI_a}{dt} = \lambda_a(N_a - I_a) - (\boldsymbol{\mu}_a + \boldsymbol{\gamma}_a)I_a \tag{15a}$$

$$\frac{dI_v}{dt} = \lambda_v(N_v - I_v) - \boldsymbol{\mu}_v I_v, \tag{15b}$$

where $\boldsymbol{\mu}_v$ and $\boldsymbol{\mu}_a$ are the natural death rates of vector species v and host species a, respectively, $\boldsymbol{\gamma}_a$ is the rate at which host a loses infectiousness (through recovery or death). $I_{a/v}$, and $N_{a/v}$ are the infected and total population sizes in host species a and vector species v, respectively, and λ_a and λ_v are the forces of infection acting host species a and vector species v,

respectively. The population size is assumed to be constant, so that the dynamics of the susceptible classes $S_a = N_a - I_a$ and $S_v = N_v - I_v$ are fully described by the behaviour of the infected classes $I_{a/v}$.

Forces of infection

The forces of infection can be written as

$$\lambda_a = \sum_{\text{vector species } v} b_{av}\boldsymbol{\tau}_v \boldsymbol{f}_{av} \frac{1}{N_a} I_v = \sum_{\text{vector species } v} b_{av}\boldsymbol{\tau}_v \boldsymbol{f}_{av} \frac{N_v}{N_a} \boldsymbol{i}_v \tag{16a}$$

$$\lambda_v = \sum_{\text{hosts species } a} b_{va}\boldsymbol{\tau}_v \boldsymbol{f}_{av} \frac{I_a}{N_a} = \sum_{\text{hosts species } a} b_{va}\boldsymbol{\tau}_v \boldsymbol{f}_{av} \boldsymbol{i}_a \tag{16b}$$

where b_{av} is the probability a susceptible host of species a gets infected when bitten by an infected vector of species v, b_{va} is the probability a susceptible vector of species v gets infected when biting and infected host of species a, $\boldsymbol{\tau}_v$ is the biting rate of vector v and $i_{a/v} = I_{a/v}/N_{a/v}$ the prevalence of the parasite in host species a and vector species v, respectively. The factor $\boldsymbol{f}_{av}$ is the proportion of all bites by vector v taken on species a.

These forces of infection can be interpreted as follows:

$$\lambda_a = \sum_{\text{vector species } v}$$

(probability of a bite from an infected vector of species v on a host of species a transmitting infection to the host)
(biting rate of vector species v)
(probability of a bite of vector species v being on host species a)
(probability of each host to be bitten among all hosts of species a)
(number of infected vectors of species v)

$$\lambda_v = \sum_{\text{hosts species } a}$$

(probability of a bite from a vector of species v on an infected host of species a transmitting infection)
(biting rate of vector species v)
(probability of a bite of vector species v being on host species a)
(probability of a bite on host species a being on an infected host)

The last term is asymmetric between vectors and hosts, because it is the vectors who actively seek out hosts at their biting rates.

Note that if the vector took bites randomly proportional to the population sizes of each host species, this would cancel the N_a term in Eqs. (16).

More generally, we would expect $\boldsymbol{f}_{av}$ to approach zero at least as fast as N_a if populations were to diminish, so the forces of infection would not diverge.

Dividing Eqs. (15) by the population sizes N_a and N_v, respectively, and setting the left-hand sides to zero (i.e., assuming the system is in equilibrium), we can calculate the forces of infection from the (measured) prevalences $\boldsymbol{i}_a^*$ and $\boldsymbol{i}_v^*$:

$$\lambda_a^* = \frac{\boldsymbol{i}_a^*}{1-\boldsymbol{i}_a^*}(\boldsymbol{\mu}_a + \boldsymbol{\gamma}_a) \tag{17a}$$

$$\lambda_v^* = \frac{\boldsymbol{i}_v^*}{1-\boldsymbol{i}_v^*}\boldsymbol{\mu}_v \tag{17b}$$

Linear stability analysis

Linearising the system given by Eqs. (15) around the disease-free equilibrium confirms R_0 given by Eq. (12) as invasion threshold. Numerical integration of the system around the endemic state reveals that the same value of R_0 is also a threshold for maintenance, that is the system does not have a backward bifurcation (Hadeler and van den Driessche, 1997).

Estimating transmission probabilities

We can use Eqs. (16) and (17) to estimate the transmission probabilities b_{av} and b_{va} if we assume that they depend solely on the susceptibility of the target of host or vector exposed to the parasite. In that case, we can write $b_{av} = b_a$ and $b_{va} = b_v$. The forces of infection are then

$$\lambda_a = \frac{b_a}{N_a} \sum_{\text{vector species } v} \boldsymbol{\tau}_v \boldsymbol{f}_{av} N_v \boldsymbol{i}_v \tag{18a}$$

$$\lambda_v = b_v \boldsymbol{\tau}_v \sum_{\text{hosts species } a} \boldsymbol{f}_{av} \boldsymbol{i}_a \tag{18b}$$

Equating these to Eqs. (16) yields $(n+m)$ equations for the $n+m$ transmission probabilities b_a and b_v. To solve these we would need to know the number of vectors N_v of each species v and the number of hosts N_a of each species a, which are difficult to measure. If we know the *relative* population sizes $\boldsymbol{n}_v = N_v/N^{\mathrm{V}}$ and $\boldsymbol{n}_a = N_a/N^{\mathrm{H}}$, that is the fraction of all vectors that are of species v and the fraction of all hosts that are of species a ($N^{\mathrm{H}} = \sum N_a$ being the total number of hosts and $N^{\mathrm{V}} = \sum N_v$ the total number of vectors in the system), we can rescale

$$\hat{b}_a = b_a \frac{N^{\mathrm{V}}}{N^{\mathrm{H}}} \tag{19}$$

yielding

$$\lambda_a = \frac{\hat{b}_a}{\boldsymbol{n}_a} \sum_{\text{vector species } v} \boldsymbol{\tau}_v \boldsymbol{f}_{av} \boldsymbol{n}_v \boldsymbol{i}_v \tag{20a}$$

$$\lambda_v = b_v \boldsymbol{\tau}_v \sum_{\text{hosts species } a} \boldsymbol{f}_{av} \boldsymbol{i}_a \tag{20b}$$

Assuming the system is in equilibrium, we can equate this to Eqs. (17) to get

$$\frac{\boldsymbol{i}_a^*}{1-\boldsymbol{i}_a^*}(\boldsymbol{\mu}_a + \boldsymbol{\gamma}_a) = \frac{\hat{b}_a}{\boldsymbol{n}_a} \sum_{\text{vector species } v} \boldsymbol{\tau}_v \boldsymbol{f}_{av} \boldsymbol{n}_v \boldsymbol{i}_v^* \tag{21a}$$

$$\frac{\boldsymbol{i}_v^*}{1-\boldsymbol{i}_v^*}\boldsymbol{\mu}_v = b_v \boldsymbol{\tau}_v \sum_{\text{hosts species } a} \boldsymbol{f}_{av} \boldsymbol{i}_a^* \tag{21b}$$

which are $(n+m)$ linear equations for the $(n+m)$ variables $\hat{b}_a$ and b_v. Solving for these, we get

$$\hat{b}_a = \frac{\boldsymbol{i}_a^*}{1-\boldsymbol{i}_a^*}\boldsymbol{n}_a(\boldsymbol{\mu}_a + \boldsymbol{\gamma}_a) \left(\sum_{\text{vector species } v} \boldsymbol{\tau}_v \boldsymbol{f}_{av} \boldsymbol{i}_v^* \right)^{-1} \tag{22a}$$

$$b_v = \frac{\boldsymbol{i}_v^*}{1-\boldsymbol{i}_v^*}\frac{\boldsymbol{\mu}_v}{\boldsymbol{\tau}_v} \left(\sum_{\text{hosts species } a} \boldsymbol{f}_{av} \boldsymbol{i}_a^* \right)^{-1} \tag{22b}$$

Determining reservoirs

The elements of the transmission matrix $\mathbf{T}$ are (see Eq. (2))

$$\begin{aligned} T_{va}^{\mathrm{VH}} &= \frac{\lambda_{va} N_v}{I_a} = b_v \boldsymbol{\tau}_v \boldsymbol{f}_{av} \frac{\boldsymbol{n}_v}{\boldsymbol{n}_a} \frac{N^{\mathrm{V}}}{N^{\mathrm{H}}} \\ T_{av}^{\mathrm{HV}} &= \frac{\lambda_{av} N_a}{I_v} = \hat{b}_a \boldsymbol{\tau}_v \boldsymbol{f}_{av} \frac{N^{\mathrm{H}}}{N^{\mathrm{V}}} \end{aligned} \tag{23}$$

and the non-zero elements of $\mathbf{C}$ are on the diagonal

$$\begin{aligned} C_v^{\mathrm{V}} &= -\mu_v \\ C_{aa}^{\mathrm{H}} &= -\mu_a - \gamma_a \end{aligned} \tag{24}$$

Since $\mathbf{C}$ is diagonal, the elements of $\mathbf{C}^{-1}$ are simply the inverses of the corresponding elements of $\mathbf{C}$.

The elements of $\mathbf{K}^{\mathrm{VH}}\mathbf{K}^{\mathrm{HV}}$ are then

$$\begin{aligned}\left(\mathbf{K}^{\mathrm{VH}}\mathbf{K}^{\mathrm{HV}}\right)_{vw} &= \sum_{\text{hosts species } a} K^{\mathrm{VH}}_{va} K^{\mathrm{HV}}_{aw} \\ &= b_v \boldsymbol{n}_v \sum_{\text{hosts species } a} \frac{\hat{b}_a \boldsymbol{\tau}_v \boldsymbol{\tau}_w \boldsymbol{f}_{av} \boldsymbol{f}_{aw}}{\boldsymbol{\mu}_w (\boldsymbol{\mu}_a + \gamma_a) \boldsymbol{n}_a}\end{aligned} \tag{25}$$

If there is only one vector species v, we can calculate the contribution of each host species a to R_0 to

$$\begin{aligned}K^{\mathrm{VH}}_a K^{\mathrm{HV}}_a &= \frac{b_v \hat{b}_a \boldsymbol{\tau}^2 \boldsymbol{f}_a^2 \boldsymbol{n}_v}{\boldsymbol{\mu}_v (\boldsymbol{\mu}_a + \gamma_a) \boldsymbol{n}_a} \\ &= \frac{1}{(1-\boldsymbol{i}^{\mathrm{V}*})(1-\boldsymbol{i}^*_a)} \frac{\boldsymbol{i}^*_a \boldsymbol{f}_a}{\sum_{\text{hosts species } b} \boldsymbol{i}^*_b \boldsymbol{f}_b}\end{aligned} \tag{26}$$

where we have made use of Eqs. (22), and of the fact that all fractions N_a/N_k can be replaced by the measurable $\boldsymbol{n}_a/\boldsymbol{n}_k$, the relative population sizes of two species (note that $\hat{b}_a$ contains the ratio of vectors to hosts, N^v/N^H). The basic reproductive number is (see Eq. (12))

$$R_0 = \sqrt{\sum_{\text{hosts } a} \frac{b_v \hat{b}_a \boldsymbol{\tau}^2 \boldsymbol{f}_a^2 \boldsymbol{n}_v}{\boldsymbol{\mu}_v (\boldsymbol{\mu}_a + \gamma_a) \boldsymbol{n}_a}} \tag{27}$$

which is similar to previous basic reproduction numbers given for HAT from similar models (Rogers, 1988; Artzrouni and Gouteux, 1996).

Given prevalence and biting preference, on the other hand, the basic reproduction number is

$$R_0 = \sqrt{\frac{1}{1-\boldsymbol{i}^{\mathrm{V}*}} \left(\sum_{\text{hosts species } a} \frac{\boldsymbol{i}^*_a}{1-\boldsymbol{i}^*_a} \boldsymbol{f}_a \right) \left(\sum_{\text{hosts species } a} \boldsymbol{i}^*_a \boldsymbol{f}_a \right)^{-1}} \tag{28}$$

The relative contribution of a species with low prevalence is equal to the product of prevalence and fraction of bites on that species:

$$\frac{K^{\mathrm{VH}}_a K^{\mathrm{HV}}_a}{\sum_b K^{\mathrm{VH}}_b K^{\mathrm{HV}}_b} = \frac{\boldsymbol{i}^*_a \boldsymbol{f}_a}{1-\boldsymbol{i}^*_a} = \boldsymbol{i}^*_a \boldsymbol{f}_a + o(\boldsymbol{i}^*_a)^2 \tag{29}$$

2.2 Correlated bites

Eqs. (2) contain the assumption that every bite has a chance of being on any of the species given by the set of probabilities $\boldsymbol{f}_{av}$. We will now consider a scenario in which bites are correlated in the sense that a vector having fed on a host of species a could be more likely to feed on another host of the

same species a than the other hosts species. To include this in our model, we introduce a parameter ξ that denotes the strength of this correlation.

Dynamic system

To capture the impact of correlated bites on model dynamics, we separate our vector classes I_v according to the host species a last bitten by the vector, and denote these I_{va}, the number of infected vectors of species v that have last fed on host species a. If ξ^{-1} is the average time spent feeding on a given species, the dynamical equations for the fraction of vector species v currently feeding on host species a are

$$\begin{aligned}\frac{dn_{va}}{dt} &= -\xi n_{va} + \sum_{\text{hosts species } b} \xi n_{vb} p_{va} \\ &= -\xi n_{va} + \xi p_{va}\end{aligned} \tag{30}$$

where p_{va} is the probability that an uncorrelated bite of vector species v is on host species a, normalised to $\sum_a p_{va} = 1$. In equilibrium we have $n_{va} = p_{va}$. If we demand that in equilibrium n_{va} corresponds to the measured fraction $\boldsymbol{f}_{av}$ of bites a vector species v inflicts on host species a, this means that

$$p_{va} = \boldsymbol{f}_{av} \tag{31}$$

The dynamical equations for I_{va} are therefore

$$\frac{dI_{va}}{dt} = \lambda_{va}(N_{va} - I_{va}) - \boldsymbol{\mu}_v I_{va} - \xi I_{va} + \sum_{\text{hosts species } b} \xi I_{vb} \boldsymbol{f}_{av} \tag{32}$$

Forces of infection

Dividing by N_{va} yields the relation between prevalence and force of infection

$$\frac{di_{va}}{dt} = \lambda_{va}(1 - i_{va}) - \boldsymbol{\mu}_v i_{va} - \xi i_{va} + \sum_{\text{hosts species } b} \xi i^*_{vb} \frac{\boldsymbol{n}_{vb}}{\boldsymbol{n}_{va}} \boldsymbol{f}_{av} \tag{33}$$

where we have made use of the fact that $N_{vb}/N_{va} = \boldsymbol{n}_{vb}/\boldsymbol{n}_{va}$. In equilibrium we can set $n^*_{va} = \boldsymbol{f}_{av}$ and get

$$\lambda_{va} = \frac{1}{1 - i^*_{va}} \left\{ i^*_{va}(\boldsymbol{\mu}_v + \xi) - \xi \sum_{\text{hosts species } b} i^*_{vb} \boldsymbol{f}_{bv} \right\} \tag{34}$$

Note that the prevalences i^*_{va} must always satisfy

$$\sum_{\text{hosts species } a} i^*_{va} \boldsymbol{f}_{av} = \boldsymbol{i}^*_v \tag{35}$$

Estimating transmission rates and determining reservoirs

To estimate transmission rates, we use the same procedure as above. Replacing Eq. (17b) with Eq. (34) and Eq. (20b) with

$$\lambda_{va} = b_v \boldsymbol{\tau}_v \boldsymbol{f}_{av} \boldsymbol{i}_{va} \tag{36}$$

yields $(n + nm)$ equations that, in combination with the m Eqs. (35), can be solved to yield the $(n + nm + m)$ variables $\hat{b}_a$, i^*_{va} and b_v, given a biting correlation ξ. From these, host species contributions can be determined as outlined above.

Correlated bites by group

Another hypothesis would have biting correlations occur not by individual species but groups of species. For example, a fly biting any domestic animal could be more likely to bite any other domestic animal (but not necessarily of the same species) than randomly. To reflect this in the framework introduced above, we replace index i representing individual species in Eq. (30) by an index representing a whole subset of species. For example, we could have three such groups (Humans – Domestic animals – Wild animals) or two (Humans/Domestic animals – Wild animals). If G is a group of species, Eqs. (30) become

$$\begin{aligned} \frac{dn_{Gj}}{dt} &= -\xi n_{Gj} + \sum_{\text{groups } H} \xi n_{Hv} p_{Gv} \\ &= -\xi n_{Gv} + \xi p_{Gv} \end{aligned} \tag{37}$$

where, by the exact same argument as above, in equilibrium we have

$$n_{Gv} = p_{Gv} = \sum_{a \in G} \boldsymbol{f}_{av} \tag{38}$$

and Eq. (33) gets replaced by

$$\frac{i_{Gv}}{dt} = \lambda_{Gv}(1 - i_{Gv}) - \boldsymbol{\mu}_v i_{Gv} - \xi i_{Gv} + \sum_{\text{groups } H} \xi i_{Hv} \frac{n^V_{Hv}}{n^V_{Gv}} \boldsymbol{f}_{Gv} \tag{39}$$

where we set $\boldsymbol{f}_{Gv} = \sum_{a \in G} \boldsymbol{f}_{av}$.

Eqs. (34) and (35) get replaced by

$$\lambda_{Gv} = \frac{1}{1 - i^*_{Gv}} \left\{ i^*_{Gv}(\boldsymbol{\mu}_v + \xi) - \xi \sum_{\text{groups } H} i^*_{Hv} \boldsymbol{f}_{Hv} \right\} \tag{40}$$

$$\sum_{\text{groups } G} i^*_{Gv} \boldsymbol{f}_{Gv} = \boldsymbol{i}^*_v \tag{41}$$

and Eqs. (20) by

$$\lambda_a = \frac{\hat{b}_a}{\boldsymbol{n}_a} \sum_{\text{vector species } v} \boldsymbol{\tau}_l \frac{\boldsymbol{f}_{av}}{\sum_{b \in G(a)} \boldsymbol{f}_{bv}} \boldsymbol{n}_v \boldsymbol{i}^*_{G(a)v} \tag{42a}$$

$$\lambda_{vG} = b_v \boldsymbol{\tau}_v \frac{\sum_{a \in G} \boldsymbol{f}_{av} \boldsymbol{i}^*_a}{\sum_{a \in G} \boldsymbol{f}_{av}} \tag{42b}$$

where $G(a)$ is the group containing host a. The framework for vector switching between individual species presented above can be seen as a special case of this where $G(a) \equiv \{a\}$.

2.3 Interaction rates from habitat overlap

To reflect overlapping habitats and species distributions therein, we now extend the approach presented in Section 2.2 to group species in different ways depending on whether or to what degree they share a habitat.

To do this, we define a mixing matrix $\boldsymbol{X}$, which describes how likely a vector is to switch (and therefore transmit infection) between two species or groups of species. The elements $\boldsymbol{X}_{ab}$ could, for example, be set to 1 or 0 to reflect whether two species can be found in the same habitat or not, or fractional values to reflect the amount of overlap between habitats. We assume that a vector will never transmit between two species of which the habitats do not overlap.

We follow the same approach as in Section 2.2, but restrict jumps by vectors between species to those in the same habitat, the frequency of such a jump for a given pair of species (or groups of species) a and b being given by $\boldsymbol{X}_{ab}$.

Dynamic system

To have consecutive bites on different species governed by $\boldsymbol{X}_{ij}$, we rewrite Eq. (32) describing the dynamical system to

$$\frac{dn_{va}}{dt} = -\xi n_{va} + \sum_{\text{hosts species } b} \xi n_{vb} \boldsymbol{X}_{ab} p_{va} \tag{43}$$

Demanding that in equilibrium $n_{va} = \boldsymbol{f}_{av}$ yields

$$p_{va} = \frac{\boldsymbol{f}_{av}}{\sum_b \boldsymbol{f}_{bv}\boldsymbol{X}_{ab}} \tag{44}$$

The dynamical equations for I_{va} are

$$\frac{dI_{va}}{dt} = \lambda_{va}(N_{va} - I_{va}) - \boldsymbol{\mu}_v I_{va} - \xi I_{va} + \xi \boldsymbol{f}_{av} \frac{\sum_b I_{vb}\boldsymbol{X}_{ab}}{\sum_b \boldsymbol{f}_{bv}\boldsymbol{X}_{ab}} \tag{45}$$

Forces of infection

Dividing by N_{va} yields the relation between prevalence and force of infection

$$\frac{di_{va}}{dt} = \lambda_{va}(1 - i_{va}) - \boldsymbol{\mu}_v i_{va} - \xi i_{va} + \xi \frac{\sum_b i^*_{vb}\boldsymbol{f}_{bv}\boldsymbol{X}_{ab}}{\sum_b \boldsymbol{f}_{bv}\boldsymbol{X}_{ab}} \tag{46}$$

so that, in equilibrium, we have

$$\lambda_{va} = \frac{1}{1 - i^*_{va}} \left\{ i^*_{va}(\boldsymbol{\mu}_v + \xi) - \xi \frac{\sum_b i^*_{vb}\boldsymbol{f}_{bv}\boldsymbol{X}_{ab}}{\sum_b \boldsymbol{f}_{bv}\boldsymbol{X}_{ab}} \right\} \tag{47}$$

and the equilibrium prevalences i^*_{va} again must satisfy

$$\sum_{\text{hosts species } a} i^*_{va}\boldsymbol{f}_{av} = \boldsymbol{i}^*_v \tag{48}$$

In our case, we have the densities $\boldsymbol{n}^h_a$ (or presence/absence) of the different species in four different habitats given, a indicating the species and h the habitat. We can then estimate the interaction rate $\boldsymbol{X}_{ab}$ between species a and b to

$$X_{ab} = \frac{\sum_h n^h_a n^h_b}{\left(\sum_h n^h_a\right)\left(\sum_h n^h_b\right)}. \tag{49}$$

3 Model extensions

Two additional aspects of trypanosomiasis dynamics can be included for greater realism: the fact that there is an incubation period of the vector in the fly, and the fact that the chances of infection are significantly reduced for flies that were not infected by trypanosomes on their first bite. Here, we describe the effect these inclusions have on the simplest model of Eqs. (15). This is easily extended to the more complicated models of correlated biting and patchy dynamics.

Since neither of these model extensions yielded qualitatively different dynamics with respect to the question of animal reservoirs, we focused on the simpler model in the main manuscript.

3.1 Incubation period

We include an additional state C for incubating flies. If the average incubation period of vector v is α_v^{-1}, the dynamical system is

$$\frac{dI_a}{dt} = \lambda_a(N_a - I_a) - (\boldsymbol{\mu}_a + \boldsymbol{\gamma}_a)I_a \tag{50a}$$

$$\frac{dC_v}{dt} = \lambda_v(N_v - I_v - C_v) - \boldsymbol{\alpha}_v C_v - \boldsymbol{\mu}_v C_v \tag{50b}$$

$$\frac{dI_v}{dt} = \boldsymbol{\alpha}_v C_v - \boldsymbol{\mu}_v I_v. \tag{50c}$$

3.2 Teneral flies

We include an additional state G for flies that have not been infected on their first bite. If only teneral flies can be infected (but then remain infected for a lifetime), the dynamical system is

$$\frac{dI_a}{dt} = \lambda_a(N_a - I_a) - (\boldsymbol{\mu}_a + \boldsymbol{\gamma}_a)I_a \tag{51a}$$

$$\frac{dI_v}{dt} = \lambda_v(N_v - I_v - G_v) - \boldsymbol{\mu}_v I_v \tag{51b}$$

$$\frac{dG_v}{dt} = (\lambda_v - \boldsymbol{\tau}_v)(N_v - I_v - G_v) - \boldsymbol{\mu}_v G_v. \tag{51c}$$

References

Artzrouni, M. and Gouteux, J. P. (1996). Control strategies for sleeping sickness in Central Africa: a model-based approach. *Trop Med Int Health*, 1(6):753–764.

Diekmann, O., Heesterbeek, J. A. P., and Roberts, M. G. (2010). The construction of next-generation matrices for compartmental epidemic models. *J R Soc Interface*, 7(47):873–885.

Hadeler, K. P. and van den Driessche, P. (1997). Backward bifurcation in epidemic control. *Math Biosci*, 146(1):15–35.

Haydon, D. T., Cleaveland, S., Taylor, L. H., and Laurenson, M. K. (2002). Identifying reservoirs of infection: a conceptual and practical challenge. *Emerg Infect Dis*, 8(12):1468–1473.

Nishiura, H., Hoye, B., Klaassen, M., Bauer, S., and Heesterbeek, H. (2009). How to find natural reservoir hosts from endemic prevalence in a multi-host population: A case study of influenza in waterfowl. *Epidemics*, 1(2):118–128.

Roberts, M. G. and Heesterbeek, J. A. P. (2003). A new method for estimating the effort required to control an infectious disease. *Proc R Soc B*, 270(1522):1359–1364.

Rogers, D. J. (1988). A general model for the African trypanosomiases. *Parasitology*, 97:193–212.
