# Supplementary material for: Identifying Transmission Cycles at the Human-Animal Interface: The Role of Animal Reservoirs in Maintaining Gambiense Human African Trypanosomiasis
Source: PLoS Comput Biol. 2013 Jan 17;9(1):e1002855. doi: 10.1371/journal.pcbi.1002855 (PMC3547827; doi:10.1371/journal.pcbi.1002855)
Supplement: Text S2 — Data. Contains the gambiense HAT prevalence data in different host and vector species as well as measured biting preferences and other parameters of the gambiense HAT transmission model. (PDF) [file pcbi.1002855.s002.pdf]

Supplementary Text S2: Data

Identifying transmission cycles at the human-animal interface: The role of animal reservoirs in maintaining gambiense Human African Trypanosomiasis

Sebastian Funk, Hiroshi Nishiura, Hans Heesterbeek,
W. John Edmunds, Francesco Checchi

Contents

1 Host species **2**
1.1 HAT Prevalence (only species with $\boldsymbol{i}^* > 0$) 2
1.2 Biting preference 2
1.3 Habitat presence/absence 3
1.4 Habitat distributions 4
1.5 Natural mortality rate 5
1.6 Rate of losing infectiousness 5
1.7 Density (relative population size) 6

2 Vector species **7**
2.1 HAT prevalence 7
2.2 Natural mortality rate 7
2.3 Biting rate 7
2.4 Incubation rate 7

References **9**

Note: All rates are given per year.

1 Host species

1.1 HAT Prevalence (only species with $i^* > 0$)

Name	Scientific name	Samples	+	i^*	Source
Human	*Homo sapiens*	3641	44	0.012	Grébaut et al. (2000)[1]
Sheep		267	18	0.067	Njiokou et al. (2010)[1]
Goat		264	8	0.030	Njiokou et al. (2010)[1]
Pig		307	1	0.0033	Njiokou et al. (2010)[1]
White-eyelid mangabey	*Cercocebus torquatus*	5	1	0.20	Njiokou et al. (2006)[1]
G. white-nosed Monkey	*Cercopithecus nictitans*	80	4	0.050	Njiokou et al. (2006)[1]
Blackstriped duiker	*Cephalophus dorsalis*	16	1	0.062	Njiokou et al. (2006)[1]
Blue duiker	*Cephalophus monticola*	200	4	0.020	Njiokou et al. (2006)[1]
Brush-tailed porcupine	*Atherurus africanus*	100	2	0.020	Njiokou et al. (2006)[1]
Giant rat	*Cricetomys gambianus*	125	3	0.024	Njiokou et al. (2006)[1]
Small-spotted genet	*Genetta servalina*	8	1	0.13	Njiokou et al. (2006)[1]
Two-spotted palm civet	*Nandinia binotata*	29	2	0.069	Njiokou et al. (2006)[1]

Notes

1. see section "Data sources" in the main manuscript.

1.2 Biting preference

Name	f	spread	Source
Human	0.39	(0.19,0.58)	Simo et al. (2008)
Sheep	0.015	(0.0075,0.023)	Simo et al. (2008)
Goat	0.013	(0.0066,0.020)	Simo et al. (2008)[1]
Pig	0.45	(0.22,0.67)	Simo et al. (2008)
White-eyelid mangabey	0.013	(0.0066,0.020)	Simo et al. (2008)[1]
Greater white-nosed Monkey	0.013	(0.0066,0.020)	Simo et al. (2008)[1]
Blackstriped duiker	0.013	(0.0066,0.020)	Simo et al. (2008)[1]
Blue duiker	0.013	(0.0066,0.020)	Simo et al. (2008)[1]
Brush-tailed porcupine	0.013	(0.0066,0.020)	Simo et al. (2008)[1]
Giant rat	0.013	(0.0066,0.020)	Simo et al. (2008)[1]
Small-spotted genet	0.013	(0.0066,0.020)	Simo et al. (2008)[1]
Two-spotted palm civet	0.013	(0.0066,0.020)	Simo et al. (2008)[1]

Notes

1. Estimated by distributing the 15% unaccounted for by Simo et al. (2008) equally among all other species (but values are allowed to vary

when chosen for sampling).

2. We estimated the error to be ±50% and normalised biting preference in each sample.

1.3 Habitat presence/absence

Name	Undist. forest	Cocoa plantation	Farmland	Village-adj. forest	Source
Human	0	1	1	1	assumed
Sheep	0	0	1	0	assumed[1]
Goat	0	0	1	0	assumed[1]
Pig	0	0	1	0	assumed[1]
White-eyelid mangabey	1	0	0	0	Massussi et al. (2009)[2]
G. white-nosed Monkey	1	0	0	0	Massussi et al. (2009)
Blackstriped duiker	1	1	1	1	Massussi et al. (2009)
Blue duiker	1	1	1	1	Massussi et al. (2009)
Brush-tailed porcupine	1	1	1	1	Massussi et al. (2009)
Giant rat	1	1	1	1	Massussi et al. (2009)
Small-spotted genet	1	1	1	1	assumed
Two-spotted palm civet	1	0	1	0	Massussi et al. (2009)

Notes

1. Domestic animals were assumed to be present only on farmland.

2. Estimated from the other primates.

1.4 Habitat distributions

Name	Undist. forest	spread	Cocoa plantation	spread	Farmland	spread
Human	0	(0,0)	7	(5.6,8.4)	7	(5.6,8.4)
Sheep	0	(0,0)	0	(0,0)	1.3	(1.0,0.9)
Goat	0	(0,0)	0	(0,0)	1.3	(1.0,1.6)
Pig	0	(0,0)	0	(0,0)	1.5	(1.2,1.8)
White-eyelid mangabey	0.74	(0.49,0.99)	0	(0,0)	0	(0,0)
G. white-nosed Monkey	1.1	(0.8,1.4)	0	(0,0)	0	(0,0)
Blackstriped duiker	0.2	(0.15,0.25)	0.05	(0.02,0.08)	0.33	(0.11,0.55)
Blue duiker	1.8	(1.4,2.1)	0.47	(0.31,0.63)	0.49	(0.31,0.67)
Brush-tailed porcupine	7.9	(5.5,10)	2.8	(1.8,3.8)	4.6	(3.0,6.3)
Giant rat	0.51	(0.43,0.59)	0.41	(0.28,0.54)	0.44	(0.35,0.53)
Small-spotted genet	0.77	(0.66,0.88)	0.25	(0.14,0.36)	0.33	(0.24,0.42)
Two-spotted palm civet	0.09	(0.06,0.12)	0	(0,0)	0.08	(0.05,0.11)

Name	Village-adj. forest	spread	Source
Human	7	(5.6,8.4)	Massussi et al. (2009)[1]
Sheep	0	(0,0)	from sheep/human ratio (Njiokou et al., 2010)[2]
Goat	0	(0,0)	from sheep/human ratio (Njiokou et al., 2010)[2]
Pig	0	(0,0)	from sheep/human ratio (Njiokou et al., 2010)[2]
White-eyelid mangabey	0	(0,0)	Massussi et al. (2009)[3]
G. white-nosed Monkey	0	(0,0)	Massussi et al. (2009)
Blackstriped duiker	0.07	(0.03,0.11)	Massussi et al. (2009)
Blue duiker	0.46	(0.28,0.64)	Massussi et al. (2009)
Brush-tailed porcupine	0.41	(0.29,0.53)	Massussi et al. (2009)
Giant rat	0.15	(0.08,0.22)	Massussi et al. (2009)
Small-spotted genet	0.18	(0.08,0.28)	Massussi et al. (2009)
Two-spotted palm civet	0	(0,0)	Massussi et al. (2009)

Notes

1. Humans were assumed to be evenly distributed between cocoa plantations, farmland and village-adjacent forest.
2. Domestic animals were assumed to be present only on farmland.
3. Estimated from the other primates, and relative population numbers in sampling campaign.
4. Values are given in mean densities per square kilometre.
5. We estimated the error to be $\pm 20\%$.

1.5 Natural mortality rate

Name	μ	spread	Source
Human	0.019	(0.015,0.023)	Index mundi
Sheep	0.15	(0.1,0.2)	Njiokou et al. (2010)[1]
Goat	0.15	(0.1,0.2)	Njiokou et al. (2010)[1]
Pig	1	(0.66,2)	Njiokou et al. (2010)[2]
White-eyelid mangabey	0.033	(0.026,0.04)	senescence[3]
Greater white-nosed Monkey	0.032	(0.026,0.039)	senescence[3]
Blackstriped duiker	0.057	(0.046,0.069)	senescence[3]
Blue duiker	0.083	(0.067,0.1)	senescence[3]
Brush-tailed porcupine	0.043	(0.035,0,052)	senescence[3]
Giant rat	0.12	(0.10,0.14)	senescence[3]
Small-spotted genet	0.068	(0.054,0.081)	senescence[3]
Two-spotted palm civet	0.047	(0.038,0.057)	senescence[3]

Notes

1. 5-10 years lifetime.
2. "Usually slaughtered after about a year" (Njiokou et al., 2010).
3. Taken from the *senescence* database at *http://genomics.senescence.info/species/*.
4. We estimated the error to be $\pm 20\%$.

1.6 Rate of losing infectiousness

Name	γ	spread	Source
Human	0.47	(0.23,1.02)	Checchi et al. (2008)[1]
Sheep	2	(0,12)	Scott et al. (1983)
Goat	2	(0,12)	no information[1]
Pig	3.5	(2.8,4.2)	Penchenier et al. (2005)
White-eyelid mangabey	2	(0,12)	no information[2]
Greater white-nosed Monkey	2	(0,12)	no information[2]
Blackstriped duiker	2	(0,12)	no information[2]
Blue duiker	2	(0,12)	no information[2]
Brush-tailed porcupine	2	(0,12)	no information[2]
Giant rat	2	(0,12)	no information[2]
Small-spotted genet	2	(0,12)	no information[2]
Two-spotted palm civet	2	(0,12)	no information[2]

Notes

1. The lower bound assumes that people remain infectious throughout the whole of both stages of infection, the haemo-lymphatic or early

and the meningo-encephalitic or late stage. It corresponds to the sum of the upper bounds of the stage durations from Checchi et al. (2008). The upper bound assumes that people are infectious only during the early stage, as it could be argued that people in the late stage do not contribute much to the disease dynamics because the debilitating effects of the disease force them to stay indoors, possibly away from tsetse sites. It corresponds to the lower bound of the early stage duration from Checchi et al. (2008). The best estimate assumes that people remain infectious throughout the early and half of the late stage, corresponding to the best estimates from Checchi et al. (2008).

2. Since the progression of trypanosome infection is generally unknown, we tested both lifetime carriage and quick clearance of the parasite (note that this does not affect the NGM in the case of random mixing).

1.7 Density (relative population size)

Name	n	spread	Source
Human	7	(5.6,8.4)	Massussi et al. (2009)
Sheep	1.10	(0.88,1.32)	from sheep/human ratio (Njiokou et al., 2010)
Goat	1.09	(0.87,1.30)	from goat/human ratio (Njiokou et al., 2010)
Pig	1.26	(1.01,1.51)	from pig/human ratio (Njiokou et al., 2010)
White-eyelid mangabey	0.055	(0.036,0.074)	Massussi et al. (2009)[1,2]
G. white-nosed Monkey	0.31	(0.22,0.4)	Massussi et al. (2009)[1]
Blackstriped duiker	0.17	(0.11,0.23)	Massussi et al. (2009)[1]
Blue duiker	0.82	(0.69,0.95)	Massussi et al. (2009)[1]
Brush-tailed porcupine	4.13	(3.27,4.99)	Massussi et al. (2009)[1]
Giant rat	0.39	(0.34,0.44)	Massussi et al. (2009)[1]
Small-spotted genet	0.12	(0.04,0.2)	Massussi et al. (2009)[1,3]
Two-spotted palm civet	0.07	(0.05,0.09)	Massussi et al. (2009)[1]

Notes

1. Averaged over all habitat types.
2. Estimated from density of Mona Monkey (Massussi et al., 2009).
3. Estimated from density of Dark Mongoose (Massussi et al., 2009).

2 Vector species

2.1 HAT prevalence

Name	N	Pos	i^*	Source
G. p. palpalis	225	8	0.036	Farikou et al. (2010)[1]

Notes

1. Study performed in 2007/08.

2.2 Natural mortality rate

Name	μ	spread	Source
G. p. palpalis	11	(5,17)	Davis et al. (2011)

2.3 Biting rate

Name	τ	spread	Source
G. p. palpalis	135	(90,180)	Davis et al. (2011)

2.4 Incubation rate

Name	α	spread	Source
G. p. palpalis	11	(9,13)	Davis et al. (2011)

References

Checchi, F., Filipe, J. A. N., Haydon, D. T., Chandramohan, D., and Chappuis, F. (2008). Estimates of the duration of the early and late stage of gambiense sleeping sickness. *BMC Infect Dis*, 8:16.

Davis, S., Aksoy, S., and Galvani, A. (2011). A global sensitivity analysis for African sleeping sickness. *Parasitology*, 138(4):516–526.

Farikou, O., Njiokou, F., Mbida, J. A. M., Njitchouang, G. R., Djeunga, H. N., Asonganyi, T., Simarro, P. P., Cuny, G., and Geiger, A. (2010). Tripartite interactions between tsetse flies, Sodalis glossinidius and trypanosomes – an epidemiological approach in two historical human African trypanosomiasis foci in Cameroon. *Infect Genet Evol*, 10(1):115–121.

Grébaut, P., Sonné, W., Bodo, J. M., Ebo'O Eyenga, V., Binzouli, J. J., Ndong Ndoé, C., Nomo, E., Nkinin, S., Njiokou, F., Ollivier, G., Foumane, V., and Bureau, P. (2000). Aspects épidémiologiques d'un foyer de maladie du sommeil mal connu: le foyer de Bipindi au Camerou. *Bull Liais Doc OCEAC*, 33:16–22.

Massussi, J. A., Djieto-Lordon, C., Njiokou, F., Laveissière, C., and van der Ploeg, J. D. (2009). Influence of habitat and seasonal variation on wild mammal diversity and distribution with special reference to the Trypanosoma brucei gambiense host-reservoir in Bipindi (Cameroon). *Acta Tropica*, 112(3):308–315.

Njiokou, F., Laveissière, C., Simo, G., Nkinin, S., Grébaut, P., Cuny, G., and Herder, S. (2006). Wild fauna as a probable animal reservoir for Trypanosoma brucei gambiense in Cameroon. *Infect Genet Evol*, 6(2):147–153.

Njiokou, F., Nimpaye, H., Simo, G., Njitchouang, G. R., Asonganyi, T., Cuny, G., and Herder, S. (2010). Domestic animals as potential reservoir hosts of Trypanosoma brucei gambiense in sleeping sickness foci in Cameroon. *Parasite*, 17(1):61–66.

Penchenier, L., Alhadji, D., Bahébégué, S., Simo, G., Laveissière, C., and Cuny, G. (2005). Spontaneous cure of domestic pigs experimentally infected by Trypanosoma brucei gambiense. implications for the control of sleeping sickness. *Vet Parasitol*, 133(1):7–11.

Scott, C. M., Frézil, J. L., Toudic, A., and Godfrey, D. G. (1983). The sheep as a potential reservoir of human trypanosomiasis in the Republic of the Congo. *Trans R Soc Trop Med Hyg*, 77(3):397–401.

Simo, G., Njiokou, F., Mbida, J. A. M., Njitchouang, G. R., Herder, S., Asonganyi, T., and Cuny, G. (2008). Tsetse fly host preference from sleeping sickness foci in Cameroon: epidemiological implications. *Infect Genet Evol*, 8(1):34–39.
